# Neuroinflammation and stress-induced pathophysiology in major depressive disorder: mechanisms and therapeutic implications

**DOI:** 10.3389/fncel.2025.1538026

**Published:** 2025-04-23

**Authors:** Kunying Zhao, Yuxiao Zhang, Shuda Yang, Lirong Xiang, Shangpeng Wu, Junfang Dong, Huan Li, Haofei Yu, Weiyan Hu

**Affiliations:** ^1^School of Pharmaceutical Science & Yunnan Provincial Key Laboratory of Pharmacology for Natural Products, Kunming Medical University, Kunming, China; ^2^College of Modern Biomedical Industry, Kunming Medical University, Kunming, China

**Keywords:** depression, neuroinflammation, microglia, astrocytes, anti-inflammatory

## Abstract

Major depressive disorder (MDD) is one of the most common mental health conditions, characterized by pervasive and persistent low mood, low self-esteem, and a loss of interest or pleasure in activities that are typically enjoyable. Despite decades of research into the etiology and pathophysiological mechanisms of depression, the therapeutic outcomes for many individuals remain less than expected. A promising new area of research focuses on stress-induced neuroinflammatory processes, such as the excessive activation and crosstalk of microglia and astrocytes in the central nervous system under stress, as well as elevated levels of pro-inflammatory cytokines, which are closely linked to the onset and progression of depression. This review summarizes the mechanisms through which neuroinflammation induces or promotes the development of depression, and also highlights the effective roles of small molecules with anti-inflammatory activity in the treatment of MDD. Understanding the specific mechanisms through which stress-induced neuroinflammation further impacts depression, and using technologies such as single-cell RNA sequencing to elucidate the specific subtypes and interactions of microglia and astrocytes in depression, is of great importance for developing more effective therapeutic strategies for MDD.

## 1 Introduction

Depression is the most common neuropsychiatric disorder and a leading cause of disability (Clayton et al., 2024; Zeng et al., 2024). According to the World Health Organization, about 350 million people worldwide suffer from depression, and among them about 1 million people commit suicide each year (Ara, 2022). The major clinical symptoms of depression include persistent feelings of sadness, anhedonia, worthlessness, hopelessness or guilt, difficulty with thinking and decision-making, suicidal ideation, and changes in weight, appetite, and sleep (Otte et al., 2016; Nestler et al., 2002). At present, the pathogenic factors of depression include environmental factors, biological factors, psychological factors, genetic factors, etc., (Cui et al., 2024).

Currently, there are several main treatments for depression, such as (1) antidepressants, (2) evidence-based psychotherapy, (3) somatic non-drug therapies (Marwaha et al., 2023). Antidepressants are mainly classified according to their mechanism of action, and the more common types are listed below: (1) Tricyclic drugs (TCAs): Imipramine, Amitriptyline, Clomipramine, etc., (2) Monoamine oxidase inhibitors (MAOIs): Tranylcypromine, Phenelzine, Selegiline, Rasagiline, etc., (3) Serotonin reuptake inhibitors (SSRIs): Fluoxetine, paroxetine, Escitalopram, etc., (4) Norepinephrine reuptake inhibitors (NERIs): Bupropion, Reboxetine, Atomoxetine, etc., (5) Serotonin-norepinephrine reuptake inhibitors (SNRIs): Venlafaxine, Desvenlafaxine, Duloxetine, etc., (6) Norepinephrine and specific serotonergic antidepressants (NaSSAs): Mirtazapine, etc., (Ménard et al., 2016; Li and Zhang, 2020). Most treatment for depression have not achieved satisfactory clinical results, in approximately 50% of previously untreated depression patients, monotherapy with antidepressants or evidence-based psychotherapy provides some relief, but does not reverse depressive symptoms and return patients to their pre-illness state (Hengartner, 2020; Malhi and Mann, 2018). Clinical studies suggest that the poor efficacy of clinical antidepressants may be related to the complex pathogenesis of depression (Ye et al., 2023). At present, the known pathophysiological mechanisms of depression include the monoamine hypothesis, receptor hypothesis, neuroendocrine hypothesis, neuroplasticity hypothesis, inflammation hypothesis, excitatory amino acid hypothesis, and intestinal flora imbalance hypothesis (Jesulola et al., 2018; Stetler and Miller, 2011). Among these hypotheses, the neuroinflammatory hypothesis has attracted increasing attention in recent years.

Immune activation and inflammatory responses are believed to be important causes of many brain diseases, such as Parkinson’s disease, Alzheimer’s disease, and Huntington’s disease (Hurley and Tizabi, 2013; Wu et al., 2021). Ongoing studies in neurophysiology and neuropsychiatry are increasingly focusing on the relationship between neuroinflammation and depression, suggesting that the immune system is involved in the pathophysiology of depression (Troubat et al., 2021). Microglia and astrocytes are important participants in the neuroimmune response. Microglia play a crucial role in brain development by regulating neurogenesis, synapse formation and elimination, and the assembly of neuronal circuits (Kreisel et al., 2014). Astrocytes, the most abundant glial cells in the central nervous system, are fundamental in regulating normal brain function and are involved in the pathologies of psychiatric and neurodegenerative diseases. Reactive astrocytes are highly heterogeneous and play an important role in restoring homeostasis and limiting tissue damage in the central nervous system (Leng et al., 2018). However, in the presence of stress or endotoxin stimulation, overactivated microglia and astrocytes can release an excessive amount of inflammatory factors. These overproduced inflammatory factors can lead to neuronal damage and are considered to induce depression (Nettis and Pariante, 2020).

This paper summarizes the roles of different polarization phenotypes of microglia and astrocytes in stress-induced neuroinflammation and their potential mechanisms in depression. Additionally, it reviews recent research on the therapeutic potential of natural compounds with anti-inflammatory properties for treating depression. The importance of identifying specific subtypes of microglia and astrocytes, as well as effective genetic targets, for depression therapy is discussed. Furthermore, the paper explores the therapeutic potential of natural compounds in modulating these distinct phenotypes and genes in the treatment of depression.

## 2 Manuscript formatting

### 2.1 Neuroinflammation

The human immune system can be viewed as a multi-layered defense network that comprehensively protects the body from external threats and internal damage. It crucially prevents the invasion of foreign microorganisms, mitigates the pathogenicity of microorganisms within the body, inhibits the proliferation of cancer cells, and promotes the rejection of transplanted tissues (Kölliker-Frers et al., 2021; Berk et al., 2013). Immune defense includes physical barriers such as the skin, various epithelia, and blood-brain barrier. The innate immune system, which relies on leukocytes, responds to infections or tissue damage through early inflammatory reactions. The adaptive immune system, which is composed of T lymphocytes and B lymphocytes, interacts with specific antigens and forms immunological memory (Zhou et al., 2024).

Inflammatory responses play a protective role in the body. Transient inflammation in the nervous system typically occurs in response to central nervous system (CNS) injury, infection, toxin exposure, or autoimmune reactions (Sarno et al., 2021; Table 1). This response is beneficial during tissue repair and development (Heneka et al., 2018). Neuroinflammation activates innate immune molecules and cellular pathways (Parsi et al., 2024). In particular, peripheral immune cells, including monocytes, granulocytes, and dendritic cells, migrate to the brain through the blood and lymphatic systems to survey for pathogens or damage and support neurological function. Animal studies have shown that endotoxin administration triggers perivascular macrophage-derived monocytes to initiate an adaptive neuroinflammatory response, involving prostaglandins and anti-inflammatory feedback mechanisms (Serna-Rodríguez et al., 2022; Balistreri and Monastero, 2023). Furthermore, exogenous immune cells, such as lymphocytes, play a critical role in limiting damage spread, providing neuroprotection, and influencing cognitive function after brain injury (Wohleb et al., 2016).

**TABLE 1:** Abbreviations.

Abbreviations	Full name
5-HT	5-hydroxytryptamine
AHR	Aryl hydrocarbon receptor
AKT	Protein kinase B
ALKBH5	Human Alk B homolog
AMPK	Adenosine 5′-monophosphate-activated protein kinase
APN	Aminopeptidase N
ATG3	Autophagy-related protein 3
ATG5	Autophagy-related protein 5
ATP	Adenosine triphosphate
B2M	Beta-2-microglobulin
BBB	Blood-brain barrier
BDNF	Brain derived neurotrophic factor
BMAL1	Basic Helix-Loop-Helix ARNT Like 1
C1Q	Complement component C1q
C3	Complement C3
CAMKII	Calcium/calmodulin-dependent protein kinase II
CCL2	C-C motif chemokine ligand 2
CCL5	C-C motif chemokine ligand 5
CD11B	CD11 antigen-like-family-member B
CD16	Low affinity immunoglobulin gamma Fc region receptor III-A
CD206	Mannose Recepto
CD32	Low affinity immunoglobulin gamma Fc region receptor II-b
CD86	CD86 molecule
CGMP	Cyclic guanosine monophosphate
CLEC2D	C-type lectin domain family 2 member D
CLIC6	Chloride intracellular channel 6
CNS	Central nervous system
COX2	Cytochrome c oxidase subunit 2
CRH	Corticotropin releasing hormone
CRP	C-reactive protein
CRS	Chronic restraint stress
CRY2	Cryptochrome circadian regulator 2
CSDS	Chronic social defeat stress
CUMS	Chronic unpredictable mild stress
CX30	Connexin 30
CX3CL1	C-X3-C motif chemokine ligand 1
CX43	Connexin 43
CXCL1	C-X-C motif chemokine ligand 1
CXCL10	C-X-C motif chemokine ligand 10
CYSLT1R	Cysteinyl leukotriene type 1 receptor
CYT-1	Cytokinesis deficient 1
DAXX	Death Domain Associated Protein
EGR1	Early growth responsive gene-1
EGR2	Early growth responsive gene-2
EGR3	Early growth responsive gene-3
EGR4	Early growth responsive gene-4
ERK	Extracellular regulated protein kinases
FOS	Fos proto-oncogene
FOS2	FosB proto-oncogene
FOXO1	Forkhead box O1
FOXO3A	Transcription factor Forkhead box protein O3
FSTL1	Follistatin Like 1
FTO	Fat mass and obesity-associated protein
GABRA2	Gamma-aminobutyric acid type A receptor subunit alpha2
GAD67	Glutamate decarboxylase 67
GLT-1	Glucose transporter type 1
GLUN2B	NMDA receptor 2B
GM-CSF	Granulocyte-macrophage colony-stimulating factor
GPX4	Glutathione peroxidase 4
GSDMD	Gasdermin D
GSH	Glutathione
HIPK2	Homeodomain interacting protein kinase 2
HMGB1	High mobility group box 1 protein
HO-1	Heme oxygenase 1
IDO	Indoleamine 2, 3-dioxygenase
IGF-1	Insulin-like growth factor 1
IKKA /B	Inhibitory kappa B kinase α/β
IL-1	Interleukin-1
IL-10	Interleukin-10
IL-13	Interleukin-13
IL-18	Interleukin-18
IL-1A	Interleukin-1α
IL-1B	Interleukin-1β
IL-4	Interleukin-4
IL-6	Interleukin-6
IFN-A	Interferon-α
IFN-B	Interferon-β
INOS	Inducible nitric oxide synthase
IRF3	Interferon regulatory Factor 3
JAK1	Janus Kinase 1
JNK	C-JunN-terminal kinase
KCNE2	potassium voltage-gated channel subfamily E regulatory subunit 2
KCNJ13	Potassium inwardly rectifying channel subfamily J member 13
LC3B-2	Microtubule-associated protein 1 light chain 3
LPS	Lipopolysaccharide
LTP	Long term potentiation
MAFG	MAF BZIP transcription factor G
MAPK	Mitogen-activated protein kinase
MCOLIN	Mucolipin
MDA	Malondialdehyde
MDD	Major depressive disorder
METTL14	Methyltransferase 14
METTL3	Methyltransferase 3
MKP-1	Mitogen-activated protein kinase phosphatase-1
MTNR1B	Melatonin receptor 1B
MTROS	Mitochondrial reactive oxygen species
MYD88	Myeloid differentiation primary response 88
NF-K B	Nuclear factor kappa-B
NLRC5	NLR family CARD domain containing 5
NLRP3	NOD-like receptor thermal protein domain associated protein 3
NMDA	N-methyl-D-aspartic acid receptor
NO	Nitric oxide
NOS2	Nitric oxide synthase 2
NR2C	Nuclear receptor subfamily 2 group C
NR4A2	Nuclear receptor subfamily 4, group A, member 2
NRF2	Nuclear factor erythroid 2-related factor 2
OGT	O-linked N-acetylglucosamine transferase
OPN	Osteopontin
ORAI1	Calcium release-activated calcium modulator 1
PDCD4	Programmed cell death factor 4
PER2	Period circadian regulator 2
PGC-1A	Peroxisome proliferator-activated receptor gamma coactivator 1α
PI3K	Phosphatidylinositol-3-kinase
PPARΓ	Peroxisome proliferator-activated receptor γ
PPP1R1B	Protein Phosphatase 1 Regulatory Inhibitor Subunit 1B
PRMT2	Protein arginine methyltransferase 2
PRMT3	Protein arginine methyltransferase 3
PRMT4	Protein arginine methyltransferase 4
PRMT6	Protein arginine methyltransferase 6
PSD-95	Postsynaptic protein-95
RAGE	The receptor of advanced glycation endproducts
ROS	Reactive oxygen species
SIRT1	Sirtuin 1
SLC7A11	Solute carrier family7member 11
SOCE	Store-operated calcium entry
SOCS3	suppressor of cytokine signaling 3
STAT1	Signal transducer and activator of transcription 1
STAT3	Signal transducer and activator of transcription 3
STING	Stimulator of interferon genes
TBK1	TANK-binding kinase 1
TFEB	Transcription factor EB
TGF-A	Transforming growth factor-α
TGF-B	Transforming growth factor-β
TLR4	Toll-like receptor 4
TLR9	Toll-like receptor 9
TNF-A	Tumor necrosis factor-α
TRAF6	Tumor necrosis factor receptor-associated factor 6
TREM1	Triggering receptor expressed on myeloid cells-1
TREM2	Triggering receptor expressed on myeloid cells-2
TRKB	Tyrosine kinase receptor B
TRPML1	Transient receptor potential mucolipin channel 1
VEGF-B	Vascular endothelial growth factor B

Chronic inflammatory responses may lead to excessive production of inflammatory factors and abnormal activation of immune cells, ultimately resulting in tissue damage. These inflammatory processes are mediated by pro-inflammatory cytokines [e.g., interleukin-1β (IL-1β), interleukin-6 (IL-6), tumor necrosis factor-α (TNF-α)], chemokines [e.g., C-C motif chemokine ligand 2 (CCL2), C-C motif chemokine ligand 5 (CCL5), C-X-C motif chemokine ligand 1 (CXCL1)], secondary messengers [e.g., nitric oxide (NO), prostaglandins], and reactive oxygen species (ROS) (Baumeister et al., 2014). Studies have shown that under pathological conditions, the permeability of the blood-brain barrier (BBB) increases, allowing peripheral cytokines to stimulate the activation of microglia and astrocytes, thereby exacerbating the inflammatory response (Abbott et al., 2010). These pro-inflammatory factors, by reducing the activity of glutamine synthetase, lead to the accumulation of glutamate, which enhances the activation of excitatory neurons, thereby triggering excitotoxicity and cell apoptosis (Alzarea et al., 2024). Meanwhile, inflammatory factors can also lead to mitochondrial dysfunction, cytochrome C release, adenosine triphosphate (ATP) depletion, free radical generation, and oxidative damage (Bhatt et al., 2023; Culmsee et al., 2018). Some therapeutic agents, such as interferon-α (IFN-α), are effective in alleviating somatic diseases; however, due to their pro-inflammatory effects, they often induce mild to moderate depressive symptoms by impairing the function of brain regions involved in emotional regulation, such as the prefrontal cortex (PFC) and the amygdala (Vignau et al., 2005; Pinto and Andrade, 2016). Important emotional regulation areas in the brain, such as the PFC and amygdala, are directly affected by the overactivation of cytokine networks. Pro-inflammatory cytokines have been reported to reduce neurotrophic supply and down-regulate neurogenesis via the brain-derived neurotrophic factor (BDNF) signaling pathway, and to debilitate hippocampal cell proliferation via the nuclear factor kappa B (NF-κB) signaling pathway. Moreover, they lead to damage to the body by increasing glutamate levels through N-methyl-D-aspartic acid receptor (NMDA) receptor activation, leading to excitotoxicity and reduced neurogenesis (Kiecolt-Glaser et al., 2015).

In recent years, the role of inflammation in neurological disorders has attracted significant attention. Research has shown that the excessive activation of pro-inflammatory cytokines, such as IL-1β, TNF-α, and IL-6, is closely associated with the pathogenesis of many central nervous system disorders, including depression (Dantzer et al., 2008; Na et al., 2014). These inflammatory mediators contribute to the development of depressive symptoms by affecting brain tissue, modulating the monoaminergic system, and triggering neurotoxic processes (Arioz et al., 2019; Shi et al., 2023; Zhou et al., 2024). Animal models established through the *in vivo* injection of lipopolysaccharide (LPS) or inflammatory factors also exhibit typical depressive symptoms, such as a decrease in aggression and curiosity (Zhang et al., 2023; Zhou et al., 2024). Inhibition of the production of inflammatory cytokines, such as IL-1β, IL-6, and TNF-α, exerts antidepressant effects (Şahin et al., 2015; Zhang et al., 2017; Cheng et al., 2016). These findings suggest that the activation of microglia is closely associated with depression, and inhibiting neuroinflammation may provide a novel therapeutic target for the treatment of depression.

#### 2.1.1 Microglia

Microglia are the primary immune cells in the brain and serve as the first line of defense. In recent years, they have garnered significant attention due to their roles in immune responses and neuroinflammation. They play a crucial role in brain development by regulating neurogenesis, synaptogenesis, synapse elimination, and the formation of neuronal circuits. Furthermore, microglia possess the ability to recognize pathogens, perform phagocytosis, present antigens, and remodel synapses (DiSabato et al., 2016).

**FIGURE 1:**
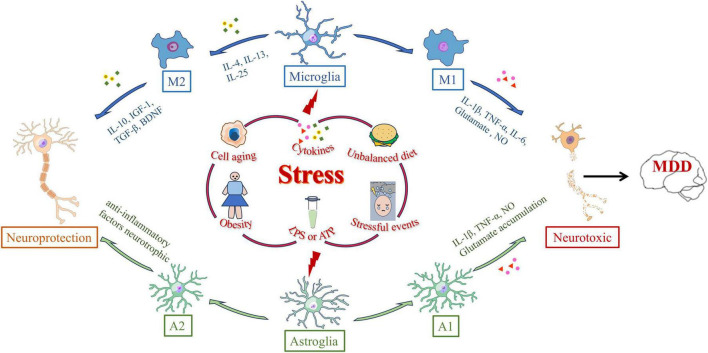
Polarization-inducing factors and dynamic properties of microglia and astrocyte. Microglia and astrocytes are polarized by many external stressors, such as prolonged stressful events, cell aging, obesity, cytokine, lipopolysaccharide (LPS) or adenosine triphosphate (ATP) stimulation, and dietary imbalance. External stress activates microglia through microglia receptors. Microglia polarized to the M1 phenotype synthesize interleukin-1β (IL-1β), tumor necrosis factor-α (TNF-α), interleukin-6 (IL-6), superoxide anion radicals, glutamate, and NO, ultimately clearing infection and repairing tissue. Cytokines such as IL-4, IL-13, or IL-25 trigger M2 activation and promote M2 microglia to release anti-inflammatory cytokines such as IL-10, insulin-like growth factor-1 (IGF-1), transforming growth factor-β (TGF-β), and brain-derived neurotrophic factor (BDNF). Astrocytes can differentiate into A1 reactive astrocytes or A2 astrocytes in response to central nervous system injury, such as injury, neurodegeneration, or infection. The expressions of complement cascade gene, IL-1β, TNF-α and NO in A1 astrocytes were significantly up-regulated. A2 astrocytes can up-regulate neurotrophic or anti-inflammatory genes, promoting the survival and growth of neurons.

Under normal conditions, resting microglia continuously monitor the surrounding environment. Upon injury or changes in the external environment, they become activated and undergo morphological changes. Microglial cells transform from a branched form to an amoeboid shape, with cell body swelling, shortened processes, increased phagocytic activity, and elevated cytokine production. This process is referred to as microglial activation (Boche et al., 2013). Microglial activation is triggered by the recognition of pattern recognition receptors (PRRs), pathogen-associated molecular patterns (PAMPs), and damage-associated molecular patterns (DAMPs). PAMPs and DAMPs interact with microglial receptors such as Toll-like receptors (TLRs) and the receptor for advanced glycation end products (RAGE), thereby initiating the synthesis and release of inflammatory mediators and promoting the transmission of inflammatory signals (Liu et al., 2023). Microglial activation can occur through two main pathways: the classical M1 activation and the selective M2 activation (Jia et al., 2021). Various factors, such as cellular aging, endotoxins, inflammatory cytokines, and ROS, can drive microglia to polarize toward the M1 phenotype. M1 microglia produce pro-inflammatory factors, including IL-1β, TNF-α, IL-6, and superoxide radicals, which help clear infections and repair tissues (Takahashi et al., 2024). In contrast, M2 activation is induced by cytokines such as interleukin-4 (IL-4) and interleukin-13 (IL-13), accompanied by the release of anti-inflammatory factors like Interleukin-10 (IL-10), insulin-like growth factor-1 (IGF 1), and transforming growth factor-β (TGF-β), which promote tissue healing, regeneration, and angiogenesis, and also repair neuronal damage (Butovsky et al., 2014; Parkhurst et al., 2013; Yi et al., 2020). It promotes healing, tissue regeneration, and angiogenesis, and can inhibit or promote the repair of neuronal injury (Wang et al., 2022; Figure 1).

However, recent research has revealed that categorizing microglia solely into M1 and M2 phenotypes is an oversimplification. Through high-throughput single-cell RNA sequencing, researchers have analyzed the RNA expression patterns of over 76,000 microglial cells from mice at different developmental stages, during aging, and in response to brain injury. The study identified at least nine distinct microglial states, which vary according to development, aging, and injury (Hammond et al., 2019). Refining the classification of microglia will help to better understand the functions, signaling mechanisms, and interactions of these subtypes with other brain cells. This, in turn, could facilitate the identification of specific microglial biomarkers for assessing human health and disease states.

Activated microglia show different responses to external stimuli, which is a double-edged sword: Acutely activated microglia usually promote tissue repair by removing invading pathogens and cell debris; Sustained microglial activation causes chronic neuroinflammation, which worsens the damage and promotes disease progression. With the deepening of the research on the pathogenesis of depression, the role of microglia in the pathogenesis of depression has also been proved in large numbers, so depression is also considered to be a microglia-related disease (microgliosis) (Yirmiya et al., 2015; Wang et al., 2022; Deng et al., 2020). Here, we summarize recent research over the past 3 years on the mechanisms through which the modulation of microglial phenotype exerts anti-inflammatory and antidepressant effects. Understanding these mechanisms is crucial for identifying new directions in the treatment and drug development for depression (Table 2).

**TABLE 2:** Microglia regulate depression-related pathways.

Types of stress	Experimental subject	Stress-induced changes in microglia	Conclusions/observations	References
CRS	C57BL/6 mice	↓STING, TBK1, IRF3, BDNF, Arg-1, ↑TNF-α, IL-1β, IL-6, CXCL10, CCL2, Iba1, iNOS	Activation of the STING/TBK1/IRF3 pathway in microglia promotes the production of IFN-β in mice under chronic restraint stress, thereby alleviating neuroinflammation and improving depressive-like behaviors, while enhancing microglial phagocytic activity	Duan et al., 2022
CSDS, LPS	C57BL/6 mice, BV2 cell	↓Nrf2, TREM2, IL-4, IL-10, Arg-1, ↑Iba1	Activation of Nrf2 can initiate the transcription of TREM2, thereby enhancing the anti-inflammatory microglial phenotype	He et al., 2022
LPS	C57BL/6 mice	↓Fos, FosB, Nr4a1, Nr4a2, Nr4a3, Egr1, Egr2, Egr3, Egr4, ↑Iba1	Nr4a2 may regulate LPS-induced depressive-like behaviors by reducing neuroinflammation, as well as improving LPS-induced microglial activation and the decreased neuronal activity of CamkII	He et al., 2023
LPS	C57BL/6 mice	↑TNF-α, IL-1α, IL-6, Iba1, IL-1β, P- NF-κB/NF-κB, P-STAT1/STAT1, P-IKKα/β/IKKα/β	APN deficiency can improve LPS-induced neuroinflammation and depressive-like behaviors by inhibiting the effect of NF-κB on BDNF/TRKB signaling	Li J. M. et al., 2022
LPS	C57BL/6 mice, BV2 cell	↓BDNF, TREM1, Copine6, Cyt-1, Per2, Cry2, Clock, ↑TNF-α, IL-6, CRP, CRH, TREM2, Bmal1	LPS induces microglial activation both *in vivo* and *in vitro*, leading to an imbalance in Bmal1 expression, which disrupts its regulation of circadian rhythm functions and impairs synaptic plasticity	Xu D. D. et al., 2024
CUMS	SD rat	↓ERK, p38, ↑MKP-1, TNF-α, IL-1β, IL-6, Iba1, -JNK	Inhibition of MKP-1 can improve ERK/p38 MAPK/JNK signaling, reversing CUMS-induced microglial activation and depressive-like behaviors in rats	Geng et al., 2024
CSDS, LPS	C57BL/6 mice, BV2 cell, HMC3 cell	↓SOCS3, P62, ↑HMGB1, RAGE, TLR4, PI3K p85, P-Akt/Akt, P-STAT3/STAT3, P-P65/P65, IL-1β, IL-6, TNF-a, Iba1, Atg3, Atg5, Beclin-1, LC3B-II	The microglial HMGB1/STAT3/p65 axis directly mediates microglial activation and autophagy in depression. Blockade of HMGB1 signaling is beneficial in improving neuroinflammation and depressive-like behaviors	Xu K. et al., 2024
LPS	BV2 cell, Primary microglia	↓PPARγ, IL-10, ↑Pdcd4, Iba1, iNOS, TNF-α, IL-1β, CCL2, B2m	Microglial Pdcd4 promotes LPS-induced neuroinflammation and depressive-like behaviors by inhibiting Daxx-mediated PPARγ nuclear translocation, thereby suppressing the expression of the anti-inflammatory cytokine IL-10	Li et al., 2024
LPS	C57BL/6 mice, BV2 cell	↓PRMT6, GPX4, GSH, ALKBH5, SLC7A11, β-catenin, ↑PRMT2, PRMT3, PRMT4, Fe^2+^, ROS, 4-HNE, MDA, TNF-α, IL-1β, IL-6, CD86, Iba1, iNOS	ALKBH5 alleviates LPS-induced ferroptosis and M1 microglial polarization by targeting the β-catenin-GPX4 axis to induce PRMT2 deficiency, ultimately exerting an antidepressant effect	Mao et al., 2024
LPS	C57BL/6 mice, BV2 cell	↓IL-4, Arg1, ↑MCPIP1, TNF-α, IL-1β, IL-6, CD16, CD32, TLR4, MyD88, TRAF6, NF-κB, Iba1, iNOS, -IL-10	MCPIP1 promotes M2 polarization of microglial cells and alleviates LPS-induced depressive-like behaviors by inhibiting the TLR4/TRAF6/NF-κB signaling pathway	Zhou et al., 2024
LPS, CUMS	C57BL/6 mice, *Nlrc5^–^/^–^*mice	↑TNF-α, IL-1β, IL-6, nuclear P65, Cleave Caspase1, P-IKK-α/β/IKK-α/β, Iba1	NLRC5 promotes the activation of classical NF-κB signaling induced by LPS by forming a complex with IKKα/β and enhancing their phosphorylation. Nlrc5 deficiency inhibits microglial activation and alleviates depressive-like behaviors in LPS and CUMS-induced mouse models of depression	Sun et al., 2023
CUMS	*FSTL1*^±^ mice	↑Iba1, TNF-α, IL-1β, IL-6, TLR4, MyD88, p-NF-κB	Partial knockdown of FSTL1 can rescue CUMS-induced microglial activation, depressive-like symptoms, and synaptic dysfunction through TLR4/MyD88/NF-κB signaling pathway	Xiao et al., 2022
LPS	C57BL/6 mice	↑Iba1, OPN, CD11b, TNF-α, IL-1β, iNOS	Blocking the expression of OPN in hippocampal microglia/macrophages of LPS-induced mice can alleviate depressive-like behaviors	Zhang et al., 2023
CUMS	*HIPK2*^–/–^ mice	↑TNF-α, IL-1β, IL-6, Iba1, p-STAT3, p-JAK1, HIPK2, Iba1, CD11b	CUMS promotes the binding and phosphorylation of HIPK2 with STAT3, thereby accelerating the M1 polarization of microglial cells, exacerbating depressive neuroinflammation, and leading to abnormal behaviors	Han et al., 2024
LPS	C57BL/6 mice	↓PI3K, Akt, BDNF, ↑TREM-1, Iba1	Inhibition of TREM-1 can alleviate LPS-induced depressive-like behaviors. The PI3K/Akt signaling pathway may be partially involved in the protective effects of TREM-1 inhibition against LPS-induced depressive-like behaviors	Fu et al., 2023
LPS	C57BL/6 mice, *FOXO3a^fl/fl^* mice	↓PPARγ, 5-HT, Arg1, CD206, ↑FOXO3a, IL-1β, Iba1, IL-6, iNOS, COX-2, NF-κB	Inhibition of FOXO3a promotes the transformation of microglial cells from the M1 to the M2 phenotype and suppresses neuroinflammation in the hippocampus, thereby alleviating LPS-induced depressive-like behaviors in mice	Wang R. et al., 2024
CUMS/CORT	C57BL/6 mice	↑IL-1β, Pro-IL-1β, Iba1, CD86, TLR9, P65, P-P65, Clec2d, P-IkBα, NLRP3, ASC, Pro-caspase-1, Cleaved caspase-1	Chronic stress leads to the activation of extracellular chromatin, which promotes ROS production in microglial cells. This triggers the NF-κB signaling pathway and activates the NLRP3 inflammasome through Clec2d and TLR9 in the mPFC	Wu et al., 2022

(↓, decrease; ↑, increase; -, no change).

#### 2.1.2 Astrocytes

Traditionally, astrocytes have been regarded as supportive cells for neurons, playing a critical role in maintaining brain homeostasis and the normal function of neurons. As the largest cell type in the CNS, astrocytes provide energy, recycle neurotransmitters, supply neurotrophic factors, and regulate synaptic formation and elimination. They also maintain the BBB and participate in immune signaling (Colombo and Farina, 2016). When the CNS undergoes damage, such as trauma, neurodegenerative diseases, or infections, astrocytes exhibit rapid changes in gene expression, morphology, and function, a response known as astrocyte reactivity (Stoklund Dittlau and Freude, 2024). Research indicates that reactive astrocytes may have detrimental effects, such as exacerbating neuroinflammation, inhibiting synaptic sprouting, or axonal growth. However, some studies suggest that A1 and A2 reactive astrocytes have beneficial roles, including anti-inflammatory effects, neuroprotection, and BBB repair (Rupareliya et al., 2023). Compared to normal astrocytes, A1 astrocytes lose many critical functions, particularly the maintenance of synaptic activity. Furthermore, A1 astrocytes significantly upregulate substances that are harmful to synapses, such as complement cascade factors, IL-1β, TNF-α, and NO (Cong et al., 2023). In contrast, A2 astrocytes can upregulate neurotrophic factors or anti-inflammatory genes, promoting neuronal survival and growth, and playing an active role in neurorepair. Astrocytes are also responsible for the uptake and metabolism of over 90% of glutamate in the brain (Mahmoud et al., 2019). When astrocytes are deficient, excessive accumulation of glutamate in the synaptic cleft may lead to excitotoxicity and an imbalance in neuronal activity (Wang et al., 2017; Figure 1).

Recent studies, however, have shown that A1 and A2 types only represent two of the potential astrocyte transcriptomes when classifying astrocytes using multi-dimensional data and co-clustering methods. Moreover, research has found that astrocytes in a healthy brain are highly diverse and perform specific roles in different CNS circuits. Reactive astrocytes are equally heterogeneous, with RNA sequencing and microarray analysis data indicating that reactive astrocytes in various disease models exhibit distinct molecular characteristics (Henrik Heiland et al., 2019).

Current research has confirmed that astrocytes are closely involved in the pathophysiology of depression. In rodent models, chronic mild stress induces overexpression of glial fibrillary acidic protein (GFAP). Increased numbers of astrocytes have also been found in the hippocampus and medial prefrontal cortex (mPFC) of patients with major depressive disorder (Wen et al., 2024; Yuan et al., 2024). Additionally, elevated levels of glutamate have been observed in the brains and cerebrospinal fluid of depression patients, and chronic stress appears to induce brain structural atrophy by disrupting the GFAP astrocytic network (Rajkowska and Stockmeier, 2013). We summarize researches conducted over the past 3 years on the mechanisms through which astrocytes mediate antidepressant effects (Table 3). These mechanistic insights may serve as potential targets for the prevention and treatment of depression (Wang J. Y. et al., 2024).

**TABLE 3:** Astrocytes regulate depression-related pathways.

Types of stress	Experimental subject	Stress-induced changes in astrocyte	Conclusions/observations	References
LPS	C57BL/6 mice, *Orai1 KO* mice	↓Orai1, SOCE	Orai1 deficiency attenuates the increase in hippocampal inflammatory markers induced by LPS in mice, as well as the inflammation-induced Ca2+ signaling in astrocytes and inhibitory neurotransmission in the hippocampus	Novakovic et al., 2023
CSDS	C57BL/6J mice, OGT-cKO mice	↑OGT, O-GlcNAc	OGT protect mPFC pyramidal neurons from glutamate-transmission deficits under social stress through the O-GlcNAcylation of GLT-1	Fan et al., 2023
CUMS	C57BL/6 J mice, *CysLT_1_R ACKO* mice	↑CysLT1R	CysLT1R knockout or knockdown in DG astrocytes improved CUMS-induced depression-like behavior in mice and restored LTP, synapse loss, PSD-95 and GluN2B levels, as well as reduced glutamate increase caused by NF-κB mediated GLT-1 reduction.	Liu X. et al., 2022
CSDS, LPS	C57BL/6J mice, *ALKBH5 KO* mice	↓METTL3, ↑ALKBH5, METTL14, FTO	Under chronic stress, astrocytic ALKBH5 preserves neuronal morphology, calcium activity, and glutamatergic transmission through m6A modification of GLT-1	Guo et al., 2024
Mtnr1b cKO^Gfap^	*Mtnr1b cKO^Gfap^* mice	↓Kcnj13, Kcne2, Gabra2, Ppp1r1b, Clic6, GAD67	The astrocyte-specific knockout in Mtnr1b cKO^Gfap^ mice results in anxiety-like behavior, which is caused by down-regulation of gamma-aminobutyric acid-ergic (GABAergic) synaptic function.	Meng et al., 2023
CUMS, SIRT6 AKO	*SIRT6 AKO* mice	↓SIRT6, ↑Cgmp	The deletion of SIRT6 in astrocytes alters purine metabolism homeostasis in the medial prefrontal cortex of mice, leading to the improvement of depressive-like behaviors in these animals	Hu et al., 2023
CUMS, LPS	C57BL/6 mice	↑IL-1β, TNF-α, MAFG, GFAP, ROS, IL-6, C3, MDA	MAFG knockdown attenuated CUMS-stimulated depression-like behaviors in mice by astrocyte-mediated neuroinflammation via restoration of HMOX1	Ye et al., 2024
LPS	Astyrocytic-*NR2C KO* mice	↑GFAP, IL-1β, TNF-α, IL-6, IL-4, IL-10, glutamate, P-JNK/JNK, P-P65/P65	Astrocytic NR2C, in conjunction with the PI3K/AKT signaling pathway, synchronously induces depression and further promotes synaptic dysfunction driven by neuroinflammation	Gao et al., 2024
CSDS	C57BL/6J mice, *TRPML1 AcKO* mice	↓MCOLIN, TRPML1, ↑SGALS3	The astrocytic TFEB-TRPML1 axis regulates depressive-like behaviors through ATP release mediated by lysosomal exocytosis	Mo et al., 2024
CUMS, CSDS	*Kir6.1 CKO* mice	↓Kir6.1, GFAP, ↑NLRP3, Caspase1, GSDMD-N, IL-1β, IL-18	The deletion of Kir6.1 in astrocytes enhances astrocytic pyroptosis and exacerbates depression through the mtROS-NLRP3-GSDMD signaling pathway	Li F. et al., 2022
MS	C57BL/6 mice, *CX43* knockdown mice	↓CX43, GFAP, CX30, GLT-1	Upregulation of CX43 can alleviate depressive-like behaviors, cognitive deficits, and astrocyte dysfunction induced by multiple sclerosis in mice	Wu et al., 2023

(↓, decrease; ↑, increase; -, no change).

#### 2.1.3 Crosstalk between microglia and astrocytes in neuroinflammation

Microglia and astrocytes play dual roles in brain diseases. They not only enhance immune responses and promote neurodegeneration, but also modulate the inflammatory responses in the central nervous system (Goshi et al., 2020). Furthermore, the interaction between astrocytes and microglia plays a critical role in neuroinflammatory responses (Olude et al., 2022).

**FIGURE 2:**
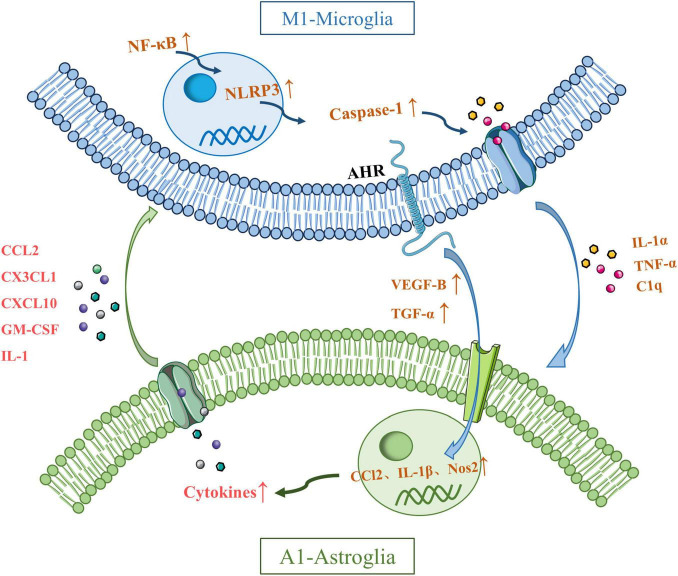
Possible mechanisms underlying crosstalk between microglia and astroglia. Interleukin-1α (IL-1α), tumor necrosis factor-α (TNF-α), and C1q secreted by activated microglia induce transcription of astrocytes to produce neurotoxic factors. Microglia can regulate the expression of CCl2, IL-1β and nitric oxide synthase 2 (NOS2) by regulating the expression of vascular endothelial growth factor B (VEGF-B) and transforming growth factor-β (TGF-α) through aryl hydrocarbon receptor (AHR). Microglial TNF-α promotes the release of glutamate from astrocytes and produces neuronal excitotoxicity. The NF-kB pathway in microglia activates the NLRP3 inflammasome, which in turn activates caspase-1 and induces the activation of A1 astrocytes. A1 astrocytes secrete CCL2, CX3CL1, CXCL10, GM-CSF, and IL-1, which in turn activate proinflammatory microglia. The deficiency of sigma-1 receptors in astrocytes leads to the activation of the NF-κB pathway, thereby amplifying the interaction between reactive astrocytes and activated microglia.

These two types of glial cells regulate inflammation in the central nervous system through the secretion of cytokines and inflammatory mediators (Kim and Son, 2021). For example, LPS-activated microglia can induce a neurotoxic phenotype in astrocytes by secreting Interleukin-1α (IL-1α), TNF-α, and complement component C1q, triggering transcriptional responses in astrocytes that lead to the production of neurotoxic factors while inhibiting phagocytic function and the expression of neurotrophic factors (Li S. et al., 2022). Moreover, aryl hydrocarbon receptor (AHR) in microglia regulates the expression of vascular endothelial growth factor B (VEGF-B) and Transforming growth factor-α (TGF-α), further promoting the expression of pro-inflammatory genes in astrocytes, such as CCL2, IL-1β, and nitric oxide synthase 2 (NOS2) (Ge et al., 2021). The production of TNF-α enhances the release of glutamate from astrocytes, thereby increasing neuronal excitotoxicity. Studies have also shown that NF-κB signaling in microglia activates the NOD-like receptor thermal protein domain associated protein 3 (NLRP3) inflammasome, which in turn triggers A1-type astrocytes through caspase-1 activation. A1 astrocytes secrete factors such as CCL2, C-X3-C motif chemokine ligand 1 (CX3CL1), C-X-C motif chemokine ligand 10 (CXCL10), granulocyte-macrophage colony-stimulating factor (GM-CSF), and interleukin-1 (IL-1), which in turn activate pro-inflammatory microglia (Linnerbauer et al., 2020; Jha et al., 2019). Furthermore, the deficiency of sigma-1 receptors in astrocytes leads to the activation of the NF-κB pathway, thereby amplifying the interaction between reactive astrocytes and activated microglia, exacerbating neuroinflammation and triggering stress-induced neuronal apoptosis, ultimately resulting in depressive-like behavior in mice (Figure 2).

### 2.2 Stress induces neuroinflammation in depression

Stress is an external stimulus that affects both the body and mind, often manifesting as emotional responses. Research suggests that the onset of depression may be related to an individual’s ability to cope with stress (Yaribeygi et al., 2017). Studies have shown that individuals who experience significant stressful events (such as the loss of a loved one, divorce, relocation, or social failure) are at a 5–6-fold increased risk of depression within 6 months (Kessler, 1997). Extensive research has demonstrated a significant causal relationship between stressful life events and the occurrence of major depressive episodes (Du Preez et al., 2021). Acute stressors are a natural physiological response to sudden events, whereas prolonged exposure to stress may lead to neuroendocrine dysfunction and emotional blunting, which can trigger mental health issues such as anxiety and depression (Lee et al., 2021).

Stress exposure experiments in rodents have shown that stress can induce the excessive secretion of cytokines (Munhoz et al., 2008). Studies have found that stress leads to elevated levels of the cytokine IL-6 in the plasma of rodents (Xu et al., 2020; Xu K. et al., 2024). Furthermore, acute restraint stress has been shown to increase the expression of IL-1β mRNA in the hypothalamus of rats (Liu H. et al., 2022; Liu et al., 2021). These findings suggest that psychosocial stressors may play an important role in the pathophysiology of stress-related disorders, such as depression, by regulating the production of pro-inflammatory and anti-inflammatory cytokines. In addition, research has revealed that stress mediators can cross the BBB and influence the immune system. Microglial cells are considered the primary source of these cytokines, and chronic stress can alter their morphology (Yao et al., 2022). In summary, the close relationship between microglial activation and neuroinflammation has been well-established. Therefore, psychological stress may induce neuroinflammation, ultimately leading to the development of depressive-like behavior.

### 2.3 Anti-inflammatory treatment can alleviate depression

Based on the impact of various neuroinflammatory lesions on the pathogenesis of depression, exploring the mechanisms and treatment methods of depression from an anti-inflammatory perspective has become a research focus in recent years (Patil et al., 2023). It is noteworthy that some of the aforementioned marketed antidepressants may alleviate depression to some extent through anti-inflammatory effects (Eyre and Baune, 2012). The serotonin reuptake inhibitor vortioxetine can inhibit the NLRP3 inflammasome pathway through its immunomodulatory effects, thus exerting antidepressant and cognitive improvement effects (Ciani et al., 2025). Additionally, a study showed that administering 10 mg/kg of ketamine to depressed model animals significantly reduced the IL-1β levels in their hippocampus (Wang et al., 2015). This suggests that anti-inflammatory treatment for depression may be an effective strategy. Common anti-inflammatory medications, such as non-steroidal anti-inflammatory drugs (NSAIDs), have shown in a meta-analysis that these drugs can effectively treat depression in animal models when used alone or in combination with antidepressants (Bai et al., 2020). However, some studies indicate that these medications may affect the efficacy of antidepressants. These mixed results may be attributed to various experimental design factors. For instance, some studies involve middle-aged patients, while others primarily focus on younger individuals. The use of selective COX-1 and COX-2 inhibitors NSAIDs has also demonstrated varying antidepressant efficacy. Furthermore, the stage of depression in patients across different studies may contribute to the observed differences in the efficacy of NSAIDs (Eyre et al., 2015; Baune, 2017).

#### 2.3.1 Natural compounds with anti-inflammatory properties have antidepressant effects

Increasingly, studies are concentrating on the mechanisms and effects of natural products with anti-inflammatory activities in improving depressive-like behaviors. The structural diversity and broad pharmacological effects of natural products are notable characteristics that are not commonly found in synthetic antidepressants (Dai et al., 2022). Natural products can modulate neural function through various mechanisms, such as affecting receptors or regulating immune processes, thereby achieving anti-inflammatory and antidepressant effects (Noori et al., 2022).

Compound 3C is a derivative of (+)-balasubramide, an 8-metalactam compound extracted from the yellow peel leaf of the Sri Lankan plant, which has been shown to have significant anti-inflammatory effects in microglia. Further investigation of the pharmacological activity of compound 3C showed that compound 3C could improve the depressive behavior of mice with endotoxins induced neuroinflammation by promoting the anti-inflammatory activity of microglia through adenosine 5’-monophosphate-activated protein kinase (AMPK)/peroxisome proliferator-activated receptor gamma coactivator 1α (PGC-1α) signaling pathway, enhancing the expression of a variety of anti-inflammatory mediators, and inhibiting the pro-inflammatory activity of microglia (Wang et al., 2018). Astragaloside IV (AS-IV) has antioxidant, anti-inflammatory, anti-hypertensive and neuroprotective effects (Abd Elkader et al., 2021). It has been reported that AS-IV may alleviate peroxisome proliferator-activated receptor γ (PPARγ)/ axis-mediated neuroinflammation and relieve depression-like behaviors in chronic restraint stress-induced and LPS-induced mice by up-regulating PPARγ expression. Baicalin, a widely used drug, has strong anti-inflammatory, anti-oxidation and anti-apoptosis activities (Song et al., 2018). Recent studies using chronic unpredictable mild stress (CUMS)-induced and endotoxin-induced depression mice have demonstrated that baicalin can improve depressive-like behavior and neuroinflammation by inhibiting the harmful overexpression of Toll-like receptor 4 (TLR4) by inhibiting the phosphatidylinositol-3-kinase (PI3K)/protein kinase B (AKT)/forkhead Box O1 (FOXO1) pathway (Guo et al., 2019). Ginsenoside Rg1 is widely reported to have a strong neuroprotective effect (Wang et al., 2023). Further evidence suggests that Rg1 may inhibit the transcriptional activity of NF-κB by increasing anti-inflammatory and inhibiting pro-inflammatory cytokines, neurotoxic mediators, pro-apoptotic proteins and microglia activation, as well as regulating mitogen activation and sirtuin 1 (SIRT1) signaling pathways. Thus, it can reduce chronic social defeat stress (CSDS) -induced hippocampal neuroinflammation and improve adult hippocampal neurogenesis, and play an antidepressant role (Jiang et al., 2020). Many previous studies have found that pinocembrin exhibit antioxidant, anti-inflammatory and neuroprotective effects both *in vitro* and *in vivo* (Li J. M. et al., 2022). Current studies have shown that Pinocembrin can reverse CUMS-induced depression-like behaviors by acting against neuroinflammation and apoptosis through nuclear factor erythroid 2-related factor 2 (Nrf2)/heme oxygenase 1 (HO-1) and NF-κB signaling pathways (Wang et al., 2020). In addition, catalpol has been shown to have anti-inflammatory, anti-tumor and anti-oxidative effects (Liang et al., 2023). Recently, catalpol has been shown to improve depression-like behaviors in CUMS mice by alleviating oxidative stress-mediated NLRP3 inflammasome activation and neuroinflammation (Wang et al., 2021). Previous studies have shown that cinnamic acid can attenuate LPS-induced depression-like behaviors by reducing LPS-induced inflammation and oxidative stress, and ameliorating LPS-induced BDNF damage (Zhuo et al., 2022). In addition to the above related findings, recent studies have also found that Asperosaponin VI exerts antidepressant effects by inhibiting TLR4/NF-κB signaling pathway, inhibiting microglia-mediated neuroinflammation, down-regulating the expression of indoleamine 2, 3-dioxygenase (IDO) and normalizing abnormal glutamate transmission (Zhang et al., 2020).

**FIGURE 3:**
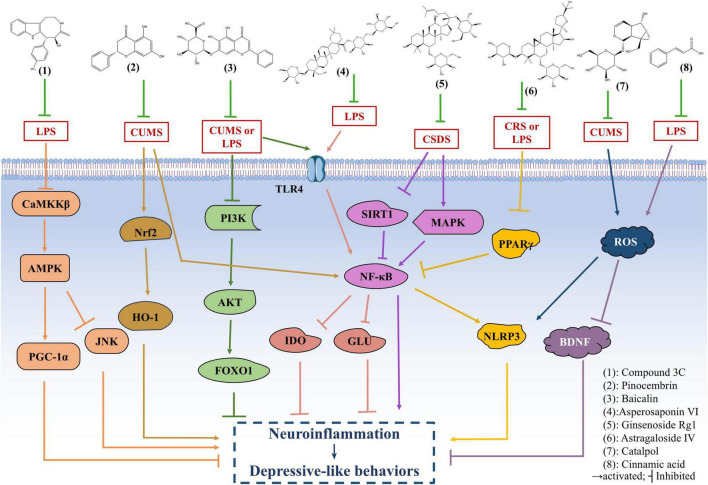
The antidepressant mechanism of natural anti-inflammatory products. Compound 3C improves the depressive behavior of mice with endotoxins induced neuroinflammation by promoting the anti-inflammatory activity of microglia through adenosine 5’-monophosphate-activated protein kinase (AMPK)/ peroxisome proliferator-activated receptor gamma coactivator 1α (PGC-1α) signaling pathway. Astragaloside IV (AS-IV) alleviates peroxisome proliferator-activated receptor γ (PPARγ)/NOD-like receptor thermal protein domain associated protein 3 (NLRP3) axis-mediated neuroinflammation and relieve depression-like behaviors in chronic unpredictable mild stress (CUMS) and lipopolysaccharide (LPS)-induced mice by up-regulating PPARγ expression. Baicalin can improve depressive-like behavior and neuroinflammation in CUMS and LPS-induced mice by inhibiting the Toll-like receptor 4 (TLR4) and phosphatidylinositol-3-kinase (PI3K)/protein kinase B (AKT)/forkhead Box O1 (FOXO1) pathway. Ginsenoside Rg1 may reduce chronic social defeat stress (CSDS)-induced depressive-like behavior in mice by inhibiting the transcriptional activity of NF-κB and regulating mitogen activation and SIRT1 signaling pathways. Pinocembrin can reverse CUMS-induced depression-like behaviors by regulating nuclear factor erythroid 2-related factor 2 (Nrf2)/heme oxygenase 1 (HO-1) and NF-kB signaling pathways. Catalpol improves depression-like behaviors in CUMS mice by alleviating oxidative stress-mediated NLRP3 inflammasome activation and neuroinflammation. Cinnamic acid can attenuate LPS-induced depression-like behaviors by reducing oxidative stress, and ameliorating LPS-induced BDNF damage. Asperosaponin VI exerts antidepressant effects by inhibiting TLR4/NF-κB signaling pathway, down-regulating the expression of indoleamine 2, 3-dioxygenase (IDO) and normalizing abnormal glutamate transmission.

By exploring the specific mechanisms and effects of these compounds in anti-inflammatory and anti-depressive effects *in vivo* and *in vitro* depression models, it lays a foundation for the pathogenesis and treatment of depression (Figure 3 and Table 4).

**TABLE 4:** Some natural anti-inflammatory products and their role in depression.

Name	Source	Pharmacological action	Mechanism	Disease model	Mode of administration	Dose	References
Compound 3C	Leaves of the Sri Lankan plant Clausena indica	Anti-apoptosis, neuroprotection, scavenging oxygen free radicals, etc.,	Promote the anti-inflammatory activity of microglia through AMPK/PGC-1α signaling pathway	C57BL/6 male mice induced by LPS	Intraperitoneal injection	1, 10 mg/kg	Wang et al., 2018
Astragaloside IV	*Astragalus membranaceus* (Fisch) Bge	Anti-oxidant, anti-inflammatory, antihypertensive, nerve protection, etc.,	Alleviate PPARγ/NLRP3 axis-mediated neuroinflammation, up-regulate PPARγ expression	C57BL/6 male mice induced by CRS or LPS	Intragastrical administration	16, 32, 64 mg/kg	Song et al., 2018
Baicalin	*Radix Scutellariae*	Anti-inflammatory, antioxidant, anti-apoptotic, neuroprotective, etc.,	Inhibit the harmful overexpression of TLR4 and the PI3K/AKT/FOXO1 pathway	ICR male mice induced by CUMS or LPS	Intragastrical administration	30, 60 mg/kg	Guo et al., 2019
Ginsenoside Rg1	Ginsenoside	Anti-oxidation, immune regulation, anti-tumor, anti-depression, anti-fatigue, etc.,	Inhibit the transcriptional activity of NF-κB, regulating mitogen activation and SIRT1 signaling pathways	C57BL/6 male mice induced by CSDS	Intragastrical administration	20, 40 mg/kg	Jiang et al., 2020
Pinocembrin	Propolis, honey	Antioxidant, anti-inflammatory, neuroprotective, etc.,	Act against neuroinflammation and apoptosis through Nrf2/HO-1 and NF-kB signaling pathways	C57BL/6 male mice induced by CUMS	Intragastrical administration	10 mg/kg	Wang et al., 2020
Catalpol	Root of *Rehmannia glutinosa Libosch*	Anti-inflammatory, anti-tumor, anti-oxidation, etc.,	Alleviate oxidative stress-mediated NLRP3 inflammasome activation and neuroinflammation	C57BL/6 male mice induced by CUMS	Intraperitoneal injection	20 mg/kg	Wang et al., 2021
Cinnamic acid	Cinnamon	Anti-inflammatory, anti-oxidation, etc.,	Reduce LPS-induced inflammation and oxidative stress, ameliorate LPS-induced BDNF damage	C57BL/6 male mice induced by LPS	Intragastrical administration	50, 100, 200 mg/kg	Zhuo et al., 2022
Asperosaponin VI	Radix Dipsaci	Anti-inflammatory, antioxidant neuroprotection, etc.,	Inhibit TLR4/NF-κB signaling pathway, microglia-mediated neuroinflammation, down-regulate the expression of IDO and normalize abnormal glutamate transmission	C57BL/6 male mice induced by LPS	Intraperitoneal injection	10, 20, 40, 80 mg/kg	Zhang et al., 2020

### 2.4 Depression increases neuroinflammation

**FIGURE 4:**
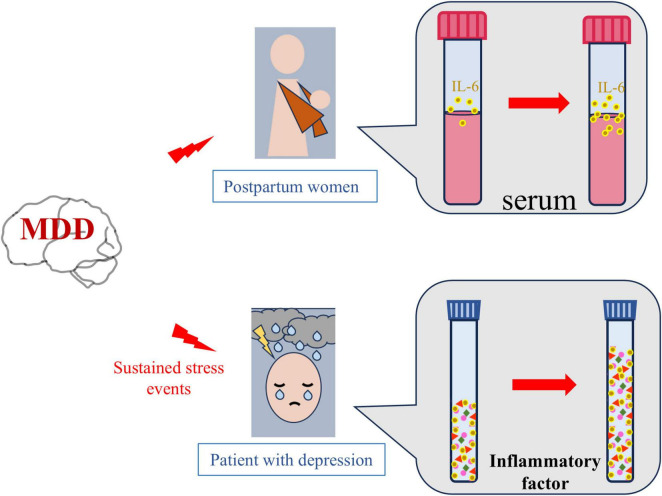
Depression increases neuroinflammation. Compared with healthy women, pregnant women with depression have higher serum levels of the inflammatory factor interleukin-6 (IL-6) after delivery. Similarly, continuous stress stimulation is more likely to lead to increased levels of inflammatory factors in patients with depression than in healthy people.

In summary, studies have found that depression and inflammation are mutually reinforcing. As mentioned above, inflammation plays a key role in the pathogenesis of depression (Slavich and Irwin, 2014). It has been mentioned in many studies related to the pathogenesis of depression that the activation of microglial in the prefrontal cortex or hippocampus and the release of pro-inflammatory factors such as IL-1β, TNF-α and IL-6 in depressed animals with stress models (Su et al., 2017; McWhirt et al., 2019). In addition, the presence of depression also triggers more cytokines in the face of stressors and pathogens (Glaser et al., 2003). Consistent with the animal literature, human studies have shown that depression triggers an inflammatory response that promotes an increase in cytokines that respond to stressors and pathogens (Rohleder and Miller, 2008; Fagundes et al., 2013). For example, in women who had just given birth, those with a lifetime history of MDD had greater increases in serum levels of IL-6 and soluble IL-6 receptors than those without a history of depression (Maes et al., 2001). Similarly, MDD patients had greater increases in inflammatory markers after stressor stimulation than non-depressed controls. A similar conclusion was reached in another study that individuals with more severe depressive symptoms were more likely to induce an increase in IL-6 in laboratory stressors. As a result, patients who are in the midst of depression are exposed to stress again, and they may continue to experience severe and recurring inflammatory responses (Pace et al., 2006; Figure 4). This suggests that depression may enhance stress response systems by promoting hyperinflammation. These findings lead to a new understanding of the complex interplay between stress, depression, and immune disorders, and the possibility that combined treatment may promote recovery and reduce relapse risk when inflammation and depression occur simultaneously. Effective depression treatment can have profound effects on mood, inflammation, and health (Kaye et al., 2000).

### 2.5 Conclusion and future perspectives

This review summarizes the relationship between long-term stress-induced neuroinflammation and the increased incidence of depression. Stress stimuli can activate the central immune system, triggering neuroinflammation, which ultimately leads to the emergence of depressive symptoms. Chronic neuroinflammation promotes the polarization of stress-induced microglia and astrocytes, stimulating the release of neurotoxic inflammatory mediators, which in turn induce symptoms such as anhedonia, memory loss, and insomnia. Furthermore, the occurrence of depression exacerbates neuroinflammation, leading to the production and release of more pro-inflammatory mediators. This review also discusses the specific mechanisms by which microglia and astrocytes modulate depression. Modulating the phenotype and function of these glial cells may provide effective strategies for the prevention and treatment of depression. Additionally, we highlight the potential of natural products with anti-inflammatory properties in improving depressive symptoms, underscoring their significant potential in the development of depression therapies. In the future, technologies such as single-cell RNA sequencing, PET, MRI, and CRISPR-Cas9 can be employed to explore the specific activation phenotypes and gene expression targets of microglia and astrocytes in depression, enabling real-time monitoring of the activation of these two glial cells. Furthermore, by targeting the specific polarization phenotypes and gene targets of microglia and astrocytes in depression, natural products that can modulate these phenotypes and act on multiple targets in combination may become a promising strategy for the prevention and treatment of depression.

## References

[B1] Abbott N. J. Patabendige A. A. Dolman D. E. Yusof S. R. Begley D. J. (2010). Structure and function of the blood-brain barrier. *Neurobiol. Dis.* 37 13–25. 10.1016/j.nbd.2009.07.030 19664713

[B2] Abd Elkader H. A. E. Abdou H. M. Khamiss O. A. Essawy A. E. (2021). Anti-anxiety and antidepressant-like effects of astragaloside IV and saponins extracted from Astragalus spinosus against the bisphenol A-induced motor and cognitive impairments in a postnatal rat model of schizophrenia. *Environ. Sci. Pollut. Res. Int.* 28 35171–35187. 10.1007/s11356-021-12927-5 33666843

[B3] Alzarea S. Khan A. Ronan P. J. Lutfy K. Rahman S. (2024). The α-7 nicotinic receptor positive allosteric modulator alleviates lipopolysaccharide induced depressive-like behavior by regulating microglial function, trophic factor, and chloride transporters in mice. *Brain Sci.* 14:290. 10.3390/brainsci14030290 38539677 PMC10968594

[B4] Ara S. K. (2022). Analysis of the prescribing pattern of antidepressants and the side effects in depression patients. *J. Family Med. Prim. Care* 11 6640–6645. 10.4103/jfmpc.jfmpc_1288_21 36993042 PMC10041272

[B5] Arioz B. I. Tastan B. Tarakcioglu E. Tufekci K. U. Olcum M. Ersoy N. (2019). Melatonin attenuates LPS-Induced acute depressive-like behaviors and microglial NLRP3 inflammasome activation through the SIRT1/Nrf2 pathway. *Front. Immunol.* 10:1511. 10.3389/fimmu.2019.01511 31327964 PMC6615259

[B6] Bai S. Guo W. Feng Y. Deng H. Li G. Nie H. (2020). Efficacy and safety of anti-inflammatory agents for the treatment of major depressive disorder: A systematic review and meta-analysis of randomised controlled trials. *J. Neurol. Neurosurg. Psychiatry* 91 21–32. 10.1136/jnnp-2019-320912 31658959

[B7] Balistreri C. R. Monastero R. (2023). Neuroinflammation and neurodegenerative diseases: How MUCH DO WE STILL NOT KNOW? *Brain Sci.* 14:19. 10.3390/brainsci14010019 38248234 PMC10812964

[B8] Baumeister D. Russell A. Pariante C. M. Mondelli V. (2014). Inflammatory biomarker profiles of mental disorders and their relation to clinical, social and lifestyle factors. *Soc. Psychiatry Psychiatr. Epidemiol.* 49 841–849. 10.1007/s00127-014-0887-z 24789456

[B9] Baune B. T. (2017). Are non-steroidal anti-inflammatory drugs clinically suitable for the treatment of symptoms in depression-associated inflammation? *Curr. Top. Behav. Neurosci.* 31 303–319. 10.1007/7854_2016_19 27405497

[B10] Berk M. Williams L. J. Jacka F. N. O’Neil A. Pasco J. A. Moylan S. (2013). So depression is an inflammatory disease, but where does the inflammation come from? *BMC Med.* 11:200. 10.1186/1741-7015-11-200 24228900 PMC3846682

[B11] Bhatt S. Dhar A. K. Samanta M. K. Suttee A. (2023). Effects of current psychotropic drugs on inflammation and immune system. *Adv. Exp. Med. Biol.* 1411 407–434. 10.1007/978-981-19-7376-5_18 36949320

[B12] Boche D. Perry V. H. Nicoll J. A. (2013). Review: Activation patterns of microglia and their identification in the human brain. *Neuropathol. Appl. Neurobiol.* 39 3–18. 10.1111/nan.12011 23252647

[B13] Butovsky O. Jedrychowski M. P. Moore C. S. Cialic R. Lanser A. J. Gabriely G. (2014). Identification of a unique TGF-β-dependent molecular and functional signature in microglia. *Nat. Neurosci.* 17 131–143. 10.1038/nn.3599 24316888 PMC4066672

[B14] Cheng Y. Pardo M. Armini R. S. Martinez A. Mouhsine H. Zagury J. F. (2016). Stress-induced neuroinflammation is mediated by GSK3-dependent TLR4 signaling that promotes susceptibility to depression-like behavior. *Brain Behav. Immun.* 53 207–222. 10.1016/j.bbi.2015.12.012 26772151 PMC4783243

[B15] Ciani M. Rigillo G. Benatti C. Pani L. Blom J. M. C. Brunello N. (2025). Time- and region-specific effect of vortioxetine on central LPS-induced transcriptional regulation of NLRP3 inflammasome. *Curr. Neuropharmacol.* 23 196–208. 10.2174/1570159x22666240705143649 39005130 PMC11793070

[B16] Clayton A. H. Suthoff E. Jain R. Kosinski M. Fridman M. Deligiannidis K. M. (2024). The magnitude and sustainability of treatment benefit of zuranolone on function and well-being as assessed by the SF-36 in adult patients with MDD and PPD: An integrated analysis of 4 randomized clinical trials. *J. Affect. Disord.* 351 904–914. 10.1016/j.jad.2024.01.268 38325605

[B17] Colombo E. Farina C. (2016). Astrocytes: Key regulators of neuroinflammation. *Trends Immunol.* 37 608–620. 10.1016/j.it.2016.06.006 27443914

[B18] Cong T. Sun Y. Zhou Y. Wu H. Li L. Chu Z. (2023). Blocking two-pore domain potassium channel TREK-1 inhibits the activation of A1-like reactive astrocyte through the NF-κB Signaling pathway in a rat model of major depressive disorder. *Neurochem. Res.* 48 1737–1754. 10.1007/s11064-023-03857-4 36670238 PMC10119044

[B19] Cui L. Li S. Wang S. Wu X. Liu Y. Yu W. (2024). Major depressive disorder: Hypothesis, mechanism, prevention and treatment. *Signal. Transduct. Target Ther.* 9:30. 10.1038/s41392-024-01738-y 38331979 PMC10853571

[B20] Culmsee C. Michels S. Scheu S. Arolt V. Dannlowski U. Alferink J. (2018). Mitochondria, microglia, and the immune system-how are they linked in affective disorders? *Front. Psychiatry* 9:739. 10.3389/fpsyt.2018.00739 30687139 PMC6333629

[B21] Dai W. Feng K. Sun X. Xu L. Wu S. Rahmand K. (2022). Natural products for the treatment of stress-induced depression: Pharmacology, mechanism and traditional use. *J. Ethnopharmacol.* 285:114692. 10.1016/j.jep.2021.114692 34742864

[B22] Dantzer R. O’Connor J. C. Freund G. G. Johnson R. W. Kelley K. W. (2008). From inflammation to sickness and depression: When the immune system subjugates the brain. *Nat. Rev. Neurosci.* 9 46–56. 10.1038/nrn2297 18073775 PMC2919277

[B23] Deng S. L. Chen J. G. Wang F. (2020). Microglia: A central player in depression. *Curr. Med. Sci.* 40 391–400. 10.1007/s11596-020-2193-1 32681244

[B24] DiSabato D. J. Quan N. Godbout J. P. (2016). Neuroinflammation: The devil is in the details. *J. Neurochem.* 139 136–153. 10.1111/jnc.13607 26990767 PMC5025335

[B25] Du Preez A. Onorato D. Eiben I. Musaelyan K. Egeland M. Zunszain P. A. (2021). Chronic stress followed by social isolation promotes depressive-like behaviour, alters microglial and astrocyte biology and reduces hippocampal neurogenesis in male mice. *Brain Behav. Immun.* 91 24–47. 10.1016/j.bbi.2020.07.015 32755644

[B26] Duan N. Zhang Y. Tan S. Sun J. Ye M. Gao H. (2022). Therapeutic targeting of STING-TBK1-IRF3 signalling ameliorates chronic stress induced depression-like behaviours by modulating neuroinflammation and microglia phagocytosis. *Neurobiol. Dis.* 169:105739. 10.1016/j.nbd.2022.105739 35470042

[B27] Eyre H. A. Air T. Proctor S. Rositano S. Baune B. T. (2015). A critical review of the efficacy of non-steroidal anti-inflammatory drugs in depression. *Prog Neuropsychopharmacol Biol. Psychiatry* 57 11–16. 10.1016/j.pnpbp.2014.10.003 25455584

[B28] Eyre H. Baune B. T. (2012). Neuroplastic changes in depression: A role for the immune system. *Psychoneuroendocrinology* 37 1397–1416. 10.1016/j.psyneuen.2012.03.019 22525700

[B29] Fagundes C. P. Glaser R. Hwang B. S. Malarkey W. B. Kiecolt-Glaser J. K. (2013). Depressive symptoms enhance stress-induced inflammatory responses. *Brain Behav. Immun.* 31 172–176. 10.1016/j.bbi.2012.05.006 22634107 PMC3518610

[B30] Fan J. Guo F. Mo R. Chen L. Y. Mo J. W. Lu C. L. (2023). O-GlcNAc transferase in astrocytes modulates depression-related stress susceptibility through glutamatergic synaptic transmission. *J. Clin. Invest.* 133:e160016. 10.1172/jci160016 36757814 PMC10065078

[B31] Fu A. Qiao F. Feng H. Luo Q. (2023). Inhibition of TREM-1 ameliorates Lipopolysaccharide-induced depressive-like behaviors by alleviating neuroinflammation in the PFC via PI3K/Akt signaling pathway. *Behav. Brain Res.* 449:114464. 10.1016/j.bbr.2023.114464 37142164

[B32] Gao R. Ali T. Liu Z. Li A. He K. Yang C. (2024). NMDAR (2C) deletion in astrocytes relieved LPS-induced neuroinflammation and depression. *Int. Immunopharmacol.* 132:111964. 10.1016/j.intimp.2024.111964 38603856

[B33] Ge T. Yao X. Zhao H. Yang W. Zou X. Peng F. (2021). Gut microbiota and neuropsychiatric disorders: Implications for neuroendocrine-immune regulation. *Pharmacol. Res.* 173:105909. 10.1016/j.phrs.2021.105909 34543739

[B34] Geng M. Shao Q. Fu J. Gu J. Feng L. Zhao L. (2024). Down-regulation of MKP-1 in hippocampus protects against stress-induced depression-like behaviors and neuroinflammation. *Transl. Psychiatry* 14:130. 10.1038/s41398-024-02846-7 38424085 PMC10904742

[B35] Glaser R. Robles T. F. Sheridan J. Malarkey W. B. Kiecolt-Glaser J. K. (2003). Mild depressive symptoms are associated with amplified and prolonged inflammatory responses after influenza virus vaccination in older adults. *Arch. Gen. Psychiatry* 60 1009–1014. 10.1001/archpsyc.60.10.1009 14557146

[B36] Goshi N. Morgan R. K. Lein P. J. Seker E. (2020). A primary neural cell culture model to study neuron, astrocyte, and microglia interactions in neuroinflammation. *J. Neuroinflammation* 17:155. 10.1186/s12974-020-01819-z 32393376 PMC7216677

[B37] Guo F. Fan J. Liu J. M. Kong P. L. Ren J. Mo J. W. (2024). Astrocytic ALKBH5 in stress response contributes to depressive-like behaviors in mice. *Nat. Commun.* 15:4347. 10.1038/s41467-024-48730-2 38773146 PMC11109195

[B38] Guo L. T. Wang S. Q. Su J. Xu L. X. Ji Z. Y. Zhang R. Y. (2019). Baicalin ameliorates neuroinflammation-induced depressive-like behavior through inhibition of toll-like receptor 4 expression via the PI3K/AKT/FoxO1 pathway. *J. Neuroinflammation* 16:95. 10.1186/s12974-019-1474-8 31068207 PMC6507025

[B39] Hammond T. R. Dufort C. Dissing-Olesen L. Giera S. Young A. Wysoker A. (2019). Single-Cell RNA sequencing of microglia throughout the mouse lifespan and in the injured brain reveals complex cell-state changes. *Immunity* 50 253–271.e6. 10.1016/j.immuni.2018.11.004 30471926 PMC6655561

[B40] Han C. Pei H. Sheng Y. Wang J. Zhou X. Li W. (2024). HIPK2 mediates M1 polarization of microglial cells via STAT3: A new mechanism of depression-related neuroinflammation. *J. Cell. Physiol.* 239:e30994. 10.1002/jcp.30994 36924038

[B41] He L. Zheng Y. Huang L. Ye J. Ye Y. Luo H. (2022). Nrf2 regulates the arginase 1(+) microglia phenotype through the initiation of TREM2 transcription, ameliorating depression-like behavior in mice. *Transl. Psychiatry* 12:459. 10.1038/s41398-022-02227-y 36316319 PMC9622811

[B42] He Y. Wang Y. Yu H. Tian Y. Chen X. Chen C. (2023). Protective effect of Nr4a2 (Nurr1) against LPS-induced depressive-like behaviors via regulating activity of microglia and CamkII neurons in anterior cingulate cortex. *Pharmacol. Res.* 191:106717. 10.1016/j.phrs.2023.106717 36948326

[B43] Heneka M. T. McManus R. M. Latz E. (2018). Inflammasome signalling in brain function and neurodegenerative disease. *Nat. Rev. Neurosci.* 19 610–621. 10.1038/s41583-018-0055-7 30206330

[B44] Hengartner M. P. (2020). How effective are antidepressants for depression over the long term? A critical review of relapse prevention trials and the issue of withdrawal confounding. *Ther. Adv. Psychopharmacol.* 10:2045125320921694. 10.1177/2045125320921694 32435449 PMC7225779

[B45] Henrik Heiland D. Ravi V. M. Behringer S. P. Frenking J. H. Wurm J. Joseph K. (2019). Tumor-associated reactive astrocytes aid the evolution of immunosuppressive environment in glioblastoma. *Nat. Commun.* 10:2541. 10.1038/s41467-019-10493-6 31186414 PMC6559986

[B46] Hu K. Chen H. Gao Y. Hua R. Shi X. Li L. (2023). Astrocytic SIRT6 is a potential anti-depression and anti-anxiety target. *Prog. Neuropsychopharmacol. Biol. Psychiatry* 123:110702. 10.1016/j.pnpbp.2022.110702 36565979

[B47] Hurley L. L. Tizabi Y. (2013). Neuroinflammation, neurodegeneration, and depression. *Neurotox Res.* 23 131–144. 10.1007/s12640-012-9348-1 22895696 PMC3751583

[B48] Jesulola E. Micalos P. Baguley I. J. (2018). Understanding the pathophysiology of depression: From monoamines to the neurogenesis hypothesis model - are we there yet? *Behav. Brain Res.* 341 79–90. 10.1016/j.bbr.2017.12.025 29284108

[B49] Jha M. K. Jo M. Kim J. H. Suk K. (2019). Microglia-astrocyte crosstalk: An intimate molecular conversation. *Neuroscientist* 25 227–240. 10.1177/1073858418783959 29931997

[B50] Jia X. Gao Z. Hu H. (2021). Microglia in depression: Current perspectives. *Sci. China Life Sci.* 64 911–925. 10.1007/s11427-020-1815-6 33068286

[B51] Jiang N. Lv J. Wang H. Huang H. Wang Q. Lu C. (2020). Ginsenoside Rg1 ameliorates chronic social defeat stress-induced depressive-like behaviors and hippocampal neuroinflammation. *Life Sci.* 252:117669. 10.1016/j.lfs.2020.117669 32298740

[B52] Kaye J. Morton J. Bowcutt M. Maupin D. (2000). Stress, depression, and psychoneuroimmunology. *J. Neurosci. Nurs.* 32 93–100. 10.1097/01376517-200004000-00005 10826295

[B53] Kessler R. C. (1997). The effects of stressful life events on depression. *Annu. Rev. Psychol.* 48 191–214. 10.1146/annurev.psych.48.1.191 9046559

[B54] Kiecolt-Glaser J. K. Derry H. M. Fagundes C. P. (2015). Inflammation: Depression fans the flames and feasts on the heat. *Am. J. Psychiatry* 172 1075–1091. 10.1176/appi.ajp.2015.15020152 26357876 PMC6511978

[B55] Kim S. Son Y. (2021). Astrocytes stimulate microglial proliferation and M2 polarization in vitro through crosstalk between astrocytes and microglia. *Int. J. Mol. Sci.* 22:8800. 10.3390/ijms22168800 34445510 PMC8396240

[B56] Kölliker-Frers R. Udovin L. Otero-Losada M. Kobiec T. Herrera M. I. Palacios J. (2021). Neuroinflammation: An integrating overview of reactive-neuroimmune cell interactions in health and disease. *Mediators Inflamm.* 2021:9999146. 10.1155/2021/9999146 34158806 PMC8187052

[B57] Kreisel T. Frank M. G. Licht T. Reshef R. Ben-Menachem-Zidon O. Baratta M. V. (2014). Dynamic microglial alterations underlie stress-induced depressive-like behavior and suppressed neurogenesis. *Mol. Psychiatry* 19 699–709. 10.1038/mp.2013.155 24342992

[B58] Lee E. H. Park J. Y. Kwon H. J. Han P. L. (2021). Repeated exposure with short-term behavioral stress resolves pre-existing stress-induced depressive-like behavior in mice. *Nat. Commun.* 12:6682. 10.1038/s41467-021-26968-4 34795225 PMC8602389

[B59] Leng L. Zhuang K. Liu Z. Huang C. Gao Y. Chen G. (2018). Menin deficiency leads to depressive-like behaviors in mice by modulating astrocyte-mediated neuroinflammation. *Neuron* 100 551–563.e7. 10.1016/j.neuron.2018.08.031 30220511

[B60] Li F. Jiang S. Y. Tian T. Li W. J. Xue Y. Du R. H. (2022). Kir6.1/K-ATP channel in astrocytes is an essential negative modulator of astrocytic pyroptosis in mouse model of depression. *Theranostics* 12 6611–6625. 10.7150/thno.77455 36185602 PMC9516231

[B61] Li J. M. Hu T. Jiang C. L. Wang W. (2022). Pinocembrin ameliorates depressive-like behaviors by regulating P2X7/TRL4 receptors expression in mouse hippocampus. *Behav. Pharmacol.* 33 301–308. 10.1097/fbp.0000000000000677 35621136

[B62] Li S. Fang Y. Zhang Y. Song M. Zhang X. Ding X. (2022). Microglial NLRP3 inflammasome activates neurotoxic astrocytes in depression-like mice. *Cell Rep.* 41:111532. 10.1016/j.celrep.2022.111532 36288697

[B63] Li W. Ali T. Zheng C. He K. Liu Z. Shah F. A. (2022). Anti-depressive-like behaviors of APN KO mice involve Trkb/BDNF signaling related neuroinflammatory changes. *Mol. Psychiatry* 27 1047–1058. 10.1038/s41380-021-01327-3 34642455

[B64] Li X. Zhang C. (2020). Comparative efficacy of nine antidepressants in treating Chinese patients with post-stroke depression: A network meta-analysis. *J. Affect. Disord.* 266 540–548. 10.1016/j.jad.2020.02.005 32056924

[B65] Li Y. Zhan B. Zhuang X. Zhao M. Chen X. Wang Q. (2024). Microglial Pdcd4 deficiency mitigates neuroinflammation-associated depression via facilitating Daxx mediated PPARγ/IL-10 signaling. *J. Neuroinflammation* 21:143. 10.1186/s12974-024-03142-3 38822367 PMC11141063

[B66] Liang X. Zhao Y. Xu T. Wang W. Sun W. Wang R. (2023). Catalpol alleviates depression by inhibiting NLRP3 inflammasome via TLR4/MAPK/NF-Kb Pathway. *Iran J. Public Health* 52 722–731. 10.18502/ijph.v52i4.12440 37551177 PMC10404318

[B67] Linnerbauer M. Wheeler M. A. Quintana F. J. (2020). Astrocyte crosstalk in CNS inflammation. *Neuron* 108 608–622. 10.1016/j.neuron.2020.08.012 32898475 PMC7704785

[B68] Liu B. Zhang Y. Yang Z. Liu M. Zhang C. Zhao Y. (2021). ω-3 DPA protected neurons from neuroinflammation by balancing microglia M1/M2 polarizations through inhibiting NF-κB/MAPK p38 signaling and activating neuron-BDNF-PI3K/AKT pathways. *Mar. Drugs* 19:587. 10.3390/md19110587 34822458 PMC8619469

[B69] Liu H. Liu L. L. Chen J. Chen Y. W. Chai Y. Liu Q. S. (2022). Muscone with attenuation of neuroinflammation and oxidative stress exerts antidepressant-like effect in mouse model of chronic restraint stress. *Oxid. Med. Cell. Longev.* 2022:3322535. 10.1155/2022/3322535 36211814 PMC9546698

[B70] Liu X. Jiang N. Zhou W. (2023). Various energetic metabolism of microglia in response to different stimulations. *Molecules* 28:4501. 10.3390/molecules28114501 37298976 PMC10254313

[B71] Liu X. Tang S. S. Liu S. M. Zeng J. Chen Z. G. Liu C. H. (2022). Deficiency of astrocyte CysLT(1)R ameliorates depression-like behaviors in mice by modulating glutamate synaptic transmission. *Neurobiol. Dis.* 175:105922. 10.1016/j.nbd.2022.105922 36371059

[B72] Maes M. Ombelet W. De Jongh R. Kenis G. Bosmans E. (2001). The inflammatory response following delivery is amplified in women who previously suffered from major depression, suggesting that major depression is accompanied by a sensitization of the inflammatory response system. *J. Affect. Disord.* 63 85–92. 10.1016/s0165-0327(00)00156-7 11246084

[B73] Mahmoud S. Gharagozloo M. Simard C. Gris D. (2019). Astrocytes maintain glutamate homeostasis in the CNS by controlling the balance between glutamate uptake and release. *Cells* 8:184. 10.3390/cells8020184 30791579 PMC6406900

[B74] Malhi G. S. Mann J. J. (2018). Depression. *Lancet* 392 2299–2312. 10.1016/s0140-6736(18)31948-2 30396512

[B75] Mao L. You J. Xie M. Hu Y. Zhou Q. (2024). Arginine methylation of β-Catenin induced by PRMT2 aggravates LPS-induced cognitive dysfunction and depression-like behaviors by promoting ferroptosis. *Mol. Neurobiol.* 61 7796–7813. 10.1007/s12035-024-04019-5 38430350

[B76] Marwaha S. Palmer E. Suppes T. Cons E. Young A. H. Upthegrove R. (2023). Novel and emerging treatments for major depression. *Lancet* 401 141–153. 10.1016/s0140-6736(22)02080-3 36535295

[B77] McWhirt J. Sathyanesan M. Sampath D. Newton S. S. (2019). Effects of restraint stress on the regulation of hippocampal glutamate receptor and inflammation genes in female C57BL/6 and BALB/c mice. *Neurobiol. Stress* 10:100169. 10.1016/j.ynstr.2019.100169 31193545 PMC6535649

[B78] Ménard C. Hodes G. E. Russo S. J. (2016). Pathogenesis of depression: Insights from human and rodent studies. *Neuroscience* 321 138–162. 10.1016/j.neuroscience.2015.05.053 26037806 PMC4664582

[B79] Meng Z. Guo S. Dong X. Wang Q. Hu D. Liu X. (2023). Astrocyte-ablation of Mtnr1b increases anxiety-like behavior in adult male mice. *J. Integr. Neurosci.* 22:154. 10.31083/j.jin2206154 38176947

[B80] Mo J. W. Kong P. L. Ding L. Fan J. Ren J. Lu C. L. (2024). Lysosomal TFEB-TRPML1 Axis in astrocytes modulates depressive-like behaviors. *Adv. Sci.* 11:e2403389. 10.1002/advs.202403389 39264289 PMC11538709

[B81] Munhoz C. D. García-Bueno B. Madrigal J. L. Lepsch L. B. Scavone C. Leza J. C. (2008). Stress-induced neuroinflammation: Mechanisms and new pharmacological targets. *Braz. J. Med. Biol. Res.* 41 1037–1046. 10.1590/s0100-879x2008001200001 19148364

[B82] Na K. S. Jung H. Y. Kim Y. K. (2014). The role of pro-inflammatory cytokines in the neuroinflammation and neurogenesis of schizophrenia. *Prog Neuropsychopharmacol Biol. Psychiatry* 48 277–286. 10.1016/j.pnpbp.2012.10.022 23123365

[B83] Nestler E. J. Barrot M. DiLeone R. J. Eisch A. J. Gold S. J. Monteggia L. M. (2002). Neurobiology of depression. *Neuron* 34 13–25. 10.1016/s0896-6273(02)00653-0 11931738

[B84] Nettis M. A. Pariante C. M. (2020). Is there neuroinflammation in depression? Understanding the link between the brain and the peripheral immune system in depression. *Int. Rev. Neurobiol.* 152 23–40. 10.1016/bs.irn.2019.12.004 32450998

[B85] Noori T. Sureda A. Sobarzo-Sánchez E. Shirooie S. (2022). The role of natural products in treatment of depressive disorder. *Curr. Neuropharmacol.* 20 929–949. 10.2174/1570159x20666220103140834 34979889 PMC9881107

[B86] Novakovic M. M. Korshunov K. S. Grant R. A. Martin M. E. Valencia H. A. Budinger G. R. S. (2023). Astrocyte reactivity and inflammation-induced depression-like behaviors are regulated by Orai1 calcium channels. *Nat. Commun.* 14:5500. 10.1038/s41467-023-40968-6 37679321 PMC10485021

[B87] Olude M. A. Mouihate A. Mustapha O. A. Farina C. Quintana F. J. Olopade J. O. (2022). Astrocytes and Microglia in Stress-Induced Neuroinflammation: The African Perspective. *Front. Immunol.* 13:795089. 10.3389/fimmu.2022.795089 35707531 PMC9190229

[B88] Otte C. Gold S. M. Penninx B. W. Pariante C. M. Etkin A. Fava M. (2016). Major depressive disorder. *Nat. Rev. Dis. Primers* 2:16065. 10.1038/nrdp.2016.65 27629598

[B89] Pace T. W. Mletzko T. C. Alagbe O. Musselman D. L. Nemeroff C. B. Miller A. H. (2006). Increased stress-induced inflammatory responses in male patients with major depression and increased early life stress. *Am. J. Psychiatry* 163 1630–1633. 10.1176/ajp.2006.163.9.1630 16946190

[B90] Parkhurst C. N. Yang G. Ninan I. Savas J. N. Yates J. R. Lafaille J. J. (2013). Microglia promote learning-dependent synapse formation through brain-derived neurotrophic factor. *Cell* 155 1596–1609. 10.1016/j.cell.2013.11.030 24360280 PMC4033691

[B91] Parsi S. Zhu C. Motlagh N. J. Kim D. Küllenberg E. G. Kim H. H. (2024). Basic science of neuroinflammation and involvement of the inflammatory response in disorders of the nervous system. *Magn. Reson. Imaging Clin. N. A.* 32 375–384. 10.1016/j.mric.2024.01.003 38555147 PMC10987041

[B92] Patil C. R. Suryakant Gawli C. Bhatt S. (2023). Targeting inflammatory pathways for treatment of the major depressive disorder. *Drug Discov. Today* 28:103697. 10.1016/j.drudis.2023.103697 37422168

[B93] Pinto E. F. Andrade C. (2016). Interferon-related depression: A primer on mechanisms, treatment, and prevention of a common clinical problem. *Curr. Neuropharmacol.* 14 743–748. 10.2174/1570159x14666160106155129 26733280 PMC5050402

[B94] Rajkowska G. Stockmeier C. A. (2013). Astrocyte pathology in major depressive disorder: Insights from human postmortem brain tissue. *Curr. Drug Targets* 14 1225–1236. 10.2174/13894501113149990156 23469922 PMC3799810

[B95] Rohleder N. Miller G. E. (2008). Acute deviations from long-term trait depressive symptoms predict systemic inflammatory activity. *Brain Behav. Immun.* 22 709–716. 10.1016/j.bbi.2007.10.012 18054461

[B96] Rupareliya V. P. Singh A. A. Butt A. M. Kumar H. (2023). The “molecular soldiers” of the CNS: Astrocytes, a comprehensive review on their roles and molecular signatures. *Eur. J. Pharmacol.* 959:176048. 10.1016/j.ejphar.2023.176048 37758010

[B97] Şahin T. D. Karson A. Balcı F. Yazır Y. Bayramgürler D. Utkan T. (2015). TNF-alpha inhibition prevents cognitive decline and maintains hippocampal BDNF levels in the unpredictable chronic mild stress rat model of depression. *Behav. Brain Res.* 292 233–240. 10.1016/j.bbr.2015.05.062 26112756

[B98] Sarno E. Moeser A. J. Robison A. J. (2021). Neuroimmunology of depression. *Adv. Pharmacol.* 91 259–292. 10.1016/bs.apha.2021.03.004 34099111 PMC8877598

[B99] Serna-Rodríguez M. F. Bernal-Vega S. de la Barquera J. A. O. Camacho-Morales A. Pérez-Maya A. A. (2022). The role of damage associated molecular pattern molecules (DAMPs) and permeability of the blood-brain barrier in depression and neuroinflammation. *J. Neuroimmunol.* 371:577951. 10.1016/j.jneuroim.2022.577951 35994946

[B100] Shi L. Xia Z. Guo J. Wang L. Peng Z. Qiu D. (2023). Maresin-1 improves LPS-induced depressive-like behavior by inhibiting hippocampal microglial activation. *J. Affect. Disord.* 328 261–272. 10.1016/j.jad.2023.02.016 36813041

[B101] Slavich G. M. Irwin M. R. (2014). From stress to inflammation and major depressive disorder: A social signal transduction theory of depression. *Psychol. Bull.* 140 774–815. 10.1037/a0035302 24417575 PMC4006295

[B102] Song M. T. Ruan J. Zhang R. Y. Deng J. Ma Z. Q. Ma S. P. (2018). Astragaloside IV ameliorates neuroinflammation-induced depressive-like behaviors in mice via the PPARγ/NF-κB/NLRP3 inflammasome axis. *Acta Pharmacol. Sin.* 39 1559–1570. 10.1038/aps.2017.208 29795356 PMC6289360

[B103] Stetler C. Miller G. E. (2011). Depression and hypothalamic-pituitary-adrenal activation: A quantitative summary of four decades of research. *Psychosom. Med.* 73 114–126. 10.1097/PSY.0b013e31820ad12b 21257974

[B104] Stoklund Dittlau K. Freude K. (2024). Astrocytes: The stars in neurodegeneration? *Biomolecules* 14:289. 10.3390/biom14030289 38540709 PMC10967965

[B105] Su W. J. Zhang Y. Chen Y. Gong H. Lian Y. J. Peng W. (2017). NLRP3 gene knockout blocks NF-κB and MAPK signaling pathway in CUMS-induced depression mouse model. *Behav. Brain Res.* 322 1–8. 10.1016/j.bbr.2017.01.018 28093255

[B106] Sun C. Shen Y. Liu P. Shen Y. Hu Y. Li P. (2023). NLRC5 deficiency reduces LPS-induced microglial activation via inhibition of NF-κB signaling and ameliorates Mice’s depressive-like behavior. *Int. J. Mol. Sci.* 24:13265. 10.3390/ijms241713265 37686068 PMC10487775

[B107] Takahashi K. Tsuji M. Nakagawasai O. Katsuyama S. Miyagawa K. Kurokawa K. (2024). Polarization to M1-type microglia in the hippocampus is involved in depression-like behavior in a mouse model of olfactory dysfunction. *Neurochem. Int.* 175:105723. 10.1016/j.neuint.2024.105723 38490486

[B108] Troubat R. Barone P. Leman S. Desmidt T. Cressant A. Atanasova B. (2021). Neuroinflammation and depression: A review. *Eur. J. Neurosci.* 53 151–171. 10.1111/ejn.14720 32150310

[B109] Vignau J. Karila L. Costisella O. Canva V. (2005). [Hepatitis C, interferon a and depression: Main physiopathologic hypothesis]. *Encephale* 31 349–357. 10.1016/s0013-7006(05)82400-5 16142050

[B110] Wang H. He Y. Sun Z. Ren S. Liu M. Wang G. (2022). Microglia in depression: An overview of microglia in the pathogenesis and treatment of depression. *J. Neuroinflammation* 19:132. 10.1186/s12974-022-02492-0 35668399 PMC9168645

[B111] Wang J. Y. Ren P. Cui L. Y. Duan J. Y. Chen H. L. Zeng Z. R. (2024). Astrocyte-specific activation of sigma-1 receptors in mPFC mediates the faster onset antidepressant effect by inhibiting NF-κB-induced neuroinflammation. *Brain Behav. Immun.* 120 256–274. 10.1016/j.bbi.2024.06.008 38852761

[B112] Wang N. Yu H. Y. Shen X. F. Gao Z. Q. Yang C. Yang J. J. (2015). The rapid antidepressant effect of ketamine in rats is associated with down-regulation of pro-inflammatory cytokines in the hippocampus. *Ups J. Med. Sci.* 120 241–248. 10.3109/03009734.2015.1060281 26220286 PMC4816884

[B113] Wang Q. Jie W. Liu J. H. Yang J. M. Gao T. M. (2017). An astroglial basis of major depressive disorder? An overview. *Glia* 65 1227–1250. 10.1002/glia.23143 28317185

[B114] Wang R. Ji L. Yuan S. Liu X. Liang Z. Chen W. (2024). Microglial forkhead box O3a deficiency attenuates LPS-induced neuro-inflammation and depressive-like behaviour through regulating the expression of peroxisome proliferator-activated receptor-γ. *Br. J. Pharmacol.* 181 3908–3925. 10.1111/bph.16474 38881194

[B115] Wang W. Wang L. Wang L. Li Y. Lan T. Wang C. (2023). Ginsenoside-Rg1 synergized with voluntary running exercise protects against glial activation and dysregulation of neuronal plasticity in depression. *Food Funct.* 14 7222–7239. 10.1039/d3fo00496a 37464840

[B116] Wang W. Zheng L. Xu L. Tu J. Gu X. (2020). Pinocembrin mitigates depressive-like behaviors induced by chronic unpredictable mild stress through ameliorating neuroinflammation and apoptosis. *Mol. Med.* 26:53. 10.1186/s10020-020-00179-x 32460706 PMC7251698

[B117] Wang Y. L. Wu H. R. Zhang S. S. Xiao H. L. Yu J. Ma Y. Y. (2021). Catalpol ameliorates depressive-like behaviors in CUMS mice via oxidative stress-mediated NLRP3 inflammasome and neuroinflammation. *Transl. Psychiatry* 11:353. 10.1038/s41398-021-01468-7 34103482 PMC8187638

[B118] Wang Y. Ruan W. Mi J. Xu J. Wang H. Cao Z. (2018). Balasubramide derivative 3C modulates microglia activation via CaMKKβ-dependent AMPK/PGC-1α pathway in neuroinflammatory conditions. *Brain Behav. Immun.* 67 101–117. 10.1016/j.bbi.2017.08.006 28803158

[B119] Wen G. Zhan X. Xu X. Xia X. Jiang S. Ren X. (2024). Ketamine improves the Glymphatic pathway by reducing the pyroptosis of hippocampal astrocytes in the chronic unpredictable mild stress model. *Mol. Neurobiol.* 61 2049–2062. 10.1007/s12035-023-03669-1 37840071

[B120] Wohleb E. S. Franklin T. Iwata M. Duman R. S. (2016). Integrating neuroimmune systems in the neurobiology of depression. *Nat. Rev. Neurosci.* 17 497–511. 10.1038/nrn.2016.69 27277867

[B121] Wu A. G. Zhou X. G. Qiao G. Yu L. Tang Y. Yan L. (2021). Targeting microglial autophagic degradation in NLRP3 inflammasome-mediated neurodegenerative diseases. *Ageing Res. Rev.* 65:101202. 10.1016/j.arr.2020.101202 33161129

[B122] Wu H. Bao H. Liu C. Zhang Q. Huang A. Quan M. (2022). Extracellular nucleosomes accelerate microglial inflammation via C-Type lectin receptor 2D and Toll-Like Receptor 9 in mPFC of mice with chronic stress. *Front. Immunol.* 13:854202. 10.3389/fimmu.2022.854202 35844599 PMC9276970

[B123] Wu X. Li L. Zhou B. Wang J. Shao W. (2023). Connexin 43 regulates astrocyte dysfunction and cognitive deficits in early life stress-treated mice. *Exp. Brain Res.* 241 1207–1214. 10.1007/s00221-023-06587-9 36939885

[B124] Xiao X. Zhang H. Ning W. Yang Z. Wang Y. Zhang T. (2022). Knockdown of FSTL1 inhibits microglia activation and alleviates depressive-like symptoms through modulating TLR4/MyD88/NF-κB pathway in CUMS mice. *Exp. Neurol.* 353:114060. 10.1016/j.expneurol.2022.114060 35367454

[B125] Xu D. D. Hou Z. Q. Xu Y. Y. Liang J. Gao Y. J. Zhang C. (2024). Potential role of Bmal1 in Lipopolysaccharide-Induced Depression-Like Behavior And Its Associated “Inflammatory Storm”. *J. Neuroimmune Pharmacol.* 19:4. 10.1007/s11481-024-10103-3 38305948

[B126] Xu K. Wang M. Wang H. Zhao S. Tu D. Gong X. (2024). HMGB1/STAT3/p65 axis drives microglial activation and autophagy exert a crucial role in chronic Stress-Induced major depressive disorder. *J. Adv. Res.* 59 79–96. 10.1016/j.jare.2023.06.003 37321346 PMC11081938

[B127] Xu X. Piao H. N. Aosai F. Zeng X. Y. Cheng J. H. Cui Y. X. (2020). Arctigenin protects against depression by inhibiting microglial activation and neuroinflammation via HMGB1/TLR4/NF-κB and TNF-α/TNFR1/NF-κB pathways. *Br. J. Pharmacol.* 177 5224–5245. 10.1111/bph.15261 32964428 PMC7589024

[B128] Yao W. Cao Q. Luo S. He L. Yang C. Chen J. (2022). Microglial ERK-NRBP1-CREB-BDNF signaling in sustained antidepressant actions of (R)-ketamine. *Mol. Psychiatry* 27 1618–1629. 10.1038/s41380-021-01377-7 34819637 PMC9095473

[B129] Yaribeygi H. Panahi Y. Sahraei H. Johnston T. P. Sahebkar A. (2017). The impact of stress on body function: A review. *Excli J.* 16 1057–1072. 10.17179/excli2017-480 28900385 PMC5579396

[B130] Ye Q. Lin S. S. Ulrich H. Tang Y. (2023). Decoupling SERT-nNOS interaction to generate fast-onset antidepressants. *Neurosci. Bull.* 39 1327–1329. 10.1007/s12264-023-01049-2 36973477 PMC10386981

[B131] Ye Y. Liang J. Xu C. Liu Y. Chen J. Zhu Y. (2024). Inhibition of HMOX1 by MAFG potentiates the development of depression-like behavior in mice associated with astrocyte-mediated neuroinflammation. *Brain Res.* 1843:149115. 10.1016/j.brainres.2024.149115 38977234

[B132] Yi S. Jiang X. Tang X. Li Y. Xiao C. Zhang J. (2020). IL-4 and IL-10 promotes phagocytic activity of microglia by up-regulation of TREM2. *Cytotechnology* 72 589–602. 10.1007/s10616-020-00409-4 32623621 PMC7450013

[B133] Yirmiya R. Rimmerman N. Reshef R. (2015). Depression as a microglial disease. *Trends Neurosci.* 38 637–658. 10.1016/j.tins.2015.08.001 26442697

[B134] Yuan Q. Lei Y. Yu K. Wu J. Xu Z. Wen C. (2024). Repetitive transcranial magnetic stimulation and fluoxetine attenuate astroglial activation and benefit behaviours in a chronic unpredictable mild stress mouse model of depression. *World J. Biol. Psychiatry* 25 82–94. 10.1080/15622975.2023.2279958 37942712

[B135] Zeng Y. Li W. Chen X. You Z. Mai S. Lan X. (2024). Mediating effect of inflammation on the relationship between sleep disruption and suicidal ideation in major depressive disorder. *J. Affect. Disord.* 352 371–378. 10.1016/j.jad.2024.02.078 38401806

[B136] Zhang J. C. Yao W. Dong C. Yang C. Ren Q. Ma M. (2017). Blockade of interleukin-6 receptor in the periphery promotes rapid and sustained antidepressant actions: A possible role of gut-microbiota-brain axis. *Transl. Psychiatry* 7:e1138. 10.1038/tp.2017.112 28556833 PMC5534942

[B137] Zhang J. Li L. Liu Q. Zhao Z. Su D. Xiao C. (2023). Gastrodin programs an Arg-1(+) microglial phenotype in hippocampus to ameliorate depression- and anxiety-like behaviors via the Nrf2 pathway in mice. *Phytomedicine* 113:154725. 10.1016/j.phymed.2023.154725 36867963

[B138] Zhang J. Yi S. Li Y. Xiao C. Liu C. Jiang W. (2020). The antidepressant effects of asperosaponin VI are mediated by the suppression of microglial activation and reduction of TLR4/NF-κB-induced IDO expression. *Psychopharmacology* 237 2531–2545. 10.1007/s00213-020-05553-5 32488348

[B139] Zhou Y. Huang Y. Ye W. Chen Z. Yuan Z. (2024). Cynaroside improved depressive-like behavior in CUMS mice by suppressing microglial inflammation and ferroptosis. *Biomed. Pharmacother.* 173:116425. 10.1016/j.biopha.2024.116425 38490155

[B140] Zhuo R. Cheng X. Luo L. Yang L. Zhao Y. Zhou Y. (2022). Cinnamic acid improved lipopolysaccharide-induced depressive-like behaviors by inhibiting neuroinflammation and oxidative stress in mice. *Pharmacology* 107 281–289. 10.1159/000520990 35325888

